# The effects of exercise referral schemes in the United Kingdom in those with cardiovascular, mental health, and musculoskeletal disorders: a preliminary systematic review

**DOI:** 10.1186/s12889-018-5868-9

**Published:** 2018-08-02

**Authors:** Nikita Rowley, Steve Mann, James Steele, Elizabeth Horton, Alfonso Jimenez

**Affiliations:** 1 Centre for Innovative Research Across the Life Course (CIRAL), Faculty of Health & Life Sciences, Coventry University, Coventry, UK; 2 Places for People Leisure, Camberley, UK; 3 ukactive Research Institute, London, UK; 4 Solent University, Southampton, UK; 5 GO fit LAB, Ingesport, Madrid, Spain

**Keywords:** Exercise referral schemes, Physical activity, Cardiovascular, Mental health, Musculoskeletal

## Abstract

**Background:**

Exercise referral schemes within clinical populations may offer benefits for inactive and sedentary individuals, and improve and aid treatment of specific health disorders. This systematic review aims to provide an overview, and examine the impact, of exercise referral schemes in patients with cardiovascular, mental health, and musculoskeletal disorders. This review focuses on populations within the United Kingdom (UK) only, with an aim to inform national exercise referral policies and guidelines.

**Method:**

Data was collected from specific sources using validated methodology through PRISMA. Systematic searches were performed using Locate, PubMed, Scopus and Pro Quest: Public Health databases. Thirteen studies met inclusion criteria set for each sub group. This included that all studies aimed to prevent, observe, or decrease ill-health relating to the disorder, participants over the age of sixteen, and health disorders and outcomes were reviewed. All studies were conducted in the UK only.

**Results:**

In the 13 articles, a variety of modes and types of exercise were utilised. One-to-one supervised exercise sessions based in a gym environment were most frequently employed. Results showed that longer length schemes (20+ weeks) produced better health outcomes, and had higher adherence to physical activity prescribed, than those of shorter length (8–12 weeks). In patients referred with cardiovascular disorders, cardiovascular-related measures showed significant decreases including blood pressure. Schemes increased physical activity levels over the length of scheme for all disorders.

**Conclusion:**

Longer length schemes (20+ weeks) improved adherence to physical activity prescribed over the course of the scheme, and could support longer term exercise adherence upon completion, however additional research on larger samples should examine this further. An implication is that schemes currently recommended in guidelines do not tailor programmes to support long term adherence to exercise, which must be addressed. There is currently a lack of research examining programmes tailored to suit the individual’s health conditions thus further research might allow providers to tailor delivery and build upon policy recommendations in the UK.

## Background

Frequent physical activity (PA) and exercise are both widely acknowledged to be effective in the prevention, management and treatment of many chronic health disorders [1–4]. PA is expressed as any bodily movement which is created by skeletal muscles that demands energy expenditure [5]. Whereas, exercise comprises of a sequence of physical activities that are repetitive and structured, with having the final intention of maintaining or improving physical fitness [6]. A body of research has documented the positive effects PA and exercise have on physiological health and psychological wellbeing [2], including enhanced mood [7], pain reduction [8], reduced risk of falls [9], decreased blood pressure (BP) and resting heart rate [9, 10], reduced depression [1, 11] and reduced anxiety [12].

Population PA levels are decreasing, creating serious repercussions for the population’s health and resulting in an epidemic of non-communicable diseases [10, 13], with an intensifying importance internationally to support the promotion of healthier lifestyles and increase in PA [14, 15]. Insufficient levels of PA are one of the major risk factors for death worldwide [16]. Within the UK, approximately 20 million adults are defined as physically inactive, increasing their risk of cardiovascular diseases (CVD), obesity, and premature death [17]. The impact of physical inactivity cost the National Health Service (NHS) £900 million in 2015 [18], which has risen to £1.2 billion in 2017 [17].

The management and treatment of many chronic disorders with PA can be built into public health pathways via exercise referral schemes (ERS). These schemes were first created in the 1990s in primary care settings to facilitate the promotion of increasing PA [19, 20]. These schemes work differently to other clinical exercise interventions as they are often employed in a non-clinical environment. This can be advantageous for individuals who may have difficulties in access to hospitals or other clinical environments which may not be local. However, they may be disadvantageous to individuals due to them typically being delivered in a leisure/gym environment, previously discovered to be a barrier to adherence in ERS [21].

ERS intended aims include increasing PA levels and thus potentially produce positive impacts on health outcomes [22]. Referrals are usually prepared by primary care professionals (including general practitioners (GPs), nurses, physiotherapists, and condition-specific specialists) to third party service providers to increase PA levels and thus improve health disorders. Participant engagement through personalised exercise programmes at leisure and sports centres are the usual path [2, 22].

Schemes habitually last 10–12 weeks within England and Ireland [2, 23]. Within Wales, the National Exercise Referral Programme lasts 16 weeks, which has been shown to be more cost-effective than schemes that conclude prior to this [24]. The National Institute for Health and Care Excellence (NICE) [22], who published the guidelines Physical Activity: Exercise Referral Schemes [Public health guideline PH54], recommend schemes last for at least 12 weeks. However, there is no emphasis on schemes to last longer than the recommended minimum of 12 weeks.

Research regarding the types of activities offered on schemes and participation rates is mixed [25]. The type and mode of physical activities which are offered in ERS include one-to-one supervised gym based exercise sessions which incorporate both cardiovascular and resistance exercises into one exercise programme, group aerobic classes, swimming, walking groups, and chair-based exercise sessions [2]. Within current ERS policy [22], there are no guidelines on the type and mode of exercise which should be encouraged within ERS. Therefore, there is demand for research to examine the various algorithms exercise which are effective in ERS, along with adherence to ERS.

At present, the literature reviewing the impact of ERS is considered to be inadequate [22, 25] due to findings revealing inconsistent and weak evidence regarding the impact of ERS on PA levels, wellbeing, quality of life or health outcomes [2, 26]. Conversely, the success of ERS is highly swayed by uptake to schemes and adherence [27]. Important evidence of the efficacy of ERS has been generated [2, 21, 26, 28], although effectiveness is influenced by the quantity of referrals who participate until completion.

There are many studies currently available which review participants with specific health disorders, however many lack strong evidence to support the effectiveness of ERS on specific health disorders [27]. Thus, NICE [22] suggested that, in addition to research examining the impact of ERS generally, further research is needed regarding the impact of ERS in improving specific health outcomes in specific populations. It is vital that the bridge between research and policy is built, to understand the role of ERS to manage specific health disorders.

There are many reasons for referral into an ERS. Health conditions can be categorised according to the ICD-10 Version: 2010 [29], which include cardiovascular, metabolic, respiratory, musculoskeletal, mental health, digestive and behavioural disorders.

ERS are often recommended in various specific health conditions including those with cardiovascular (CV), mental health (MH), and musculoskeletal (MSK) disorders. Despite different aetiologies, symptoms, and co-morbidities of these disorders, the effectiveness of ERS has often been judged based upon their overall impact in populations undertaking them, as opposed to their effectiveness of those with specific disorders, or upon specific health outcomes [2]. As such, there is a need to examine the effectiveness of ERS in this regard also. Reviewing the effectiveness of ERS in specific disorders and upon specific outcomes, could inform guidelines on management and treatment.

At present, the NHS is under pressure [30]. Referral of more patients into ERS may have the potential to reduce this burden. CV disorders affect approximately 5.9 million people within the UK, with healthcare costs estimated at £9 billion each year [31]. CV disease costs the UK economy an estimated £19 billion each year [31]. The cost of hypertension can be as high as £2040 per person, with a heart attack costing the NHS £2390 per incidence. MH disorders are one of the major causes of overall disease burden worldwide, with the most predominant MH disorder being depression [32]. The cost to the economy is estimated £105 billion a year according to the NHS [33]. MSK disorders affect approximately 23 million people in the UK, with over 30 million work days are lost each year as a result [34].

As noted, a lack of PA increases the risk of non-communicable diseases [13, 17]. For many of those disorders mentioned, patients will often visit their GP as a first point of contact. If exercise can be used as a management tool to aid health disorders, then this could impact GP visits and reduce them over time. It costs the NHS £242 per hour of patient contact [30]. To put an individual through a 12-week ERS scheme costs approximately £225. If ERS are found to be effective in improving health outcomes, then the NHS could reduce money spent on GP contact time, and invest into referring people into exercise [35].

At present, there is a lack of evidence to support the effectiveness of ERS. In their guidelines in 2014, NICE [22] suggested that this lack of evidence is a critical point for consideration, as there had been to that point a lack of progress within research to increase the evidence-base for these schemes [22]. It has been suggested that ERS stakeholders at present have conflicting and inconsistent views of the evidence which can influence funding opportunities [22, 36]. A previous systematic review of the effects of ERS on PA and improving health outcomes found that there was still uncertainty as to their effectiveness [2]. This review suggested that further research is required to separately report outcomes, and review disorder-specific populations. This systematic review aims to meet these suggestions, with an update on current research since 2011, to improve recommendations and to advance NICE policy recommendations. Thus, the main aim of this systematic review is to examine the effects of ERS in persons with CV, MH, and MSK disorders within the UK.

## Method

**Fig. 1:**
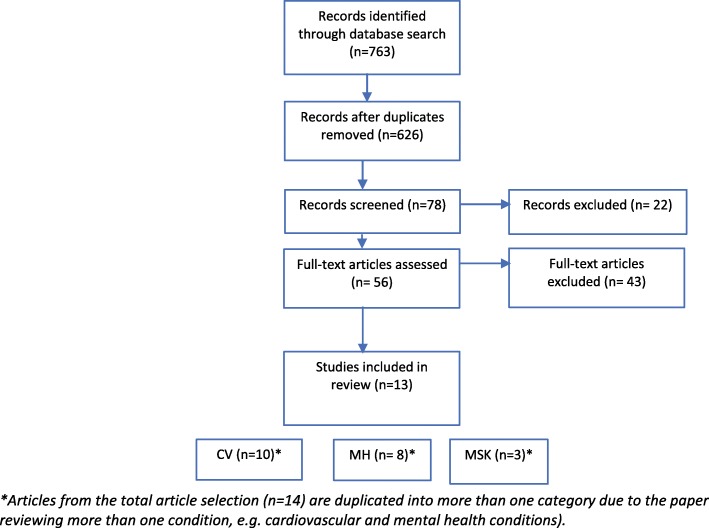
PRISMA flowchart detailing flow of studies through the review

This review follows the guidelines set out by the Preferred Reporting Items for Systematic Reviews and Meta-Analyses (PRISMA) [37]. A PRISMA flow diagram can be seen in Fig. 1.

### Search strategy

This systematic review was based on a literature search in each of the following databases: Locate, PubMed, Scopus and Pro Quest: Public Health. To maximise the specificity, initial searches used “*exercise referral schemes*” and “*adherence*”, along with sub-group specific words (“*cardiovascular*”, “*mental health*”, “*depression*”, “*anxiety*”, “*musculoskeletal*”). A title and abstract search was conducted primarily, with restrictions on publication dates (post January 2011 due to Pavey, et al. [2] reviewing for CV and MH disorder sub-groups, and an open search for the MSK disorder due to lack of research included in that review within this sub-group). During the primary search of title and abstract, one reviewer initially ruled out clearly irrelevant titles and abstracts. Articles were UK-based studies due to the potential impact of this review on changing UK ERS policies and guidelines. During the next stage, two reviewers then independently screened the remaining titles and abstracts. During the final stage, full abstracts categorised as potentially eligible for inclusion were screened by consensus of three reviewers and disagreements were resolved by consensus. Risk of bias criteria came from previous instruments which assessed the quality or risk of bias, which reduced any bias [38, 39].

### Inclusion criteria

Papers which were included met the following conditions: (a) One of the main aims being prevention, observation, or treatment of the health condition (including (1) CV disorders, (2) MH disorders, and (3) MSK disorders); (b) participants were over the age of sixteen; (c) health conditions and their outcomes were reviewed; and, (d) UK-based studies only, due to the potential of this paper possibly influencing UK ERS policy. Studies were included regardless of the study design, as the primary aim was to provide an updated review of the ERS to inform policy and guidelines; (e) utilised outcome measures which measured CV, MH and MSK conditions of participants.

### Exclusion criteria

Papers were excluded if: (a) Articles were not published in a peer reviewed journal (annual reports, editorials, systematic reviews/meta-analyses, opinions and studies available only as abstracts were also excluded); (b) If participants were below the age of sixteen, as guidelines states people must be over 16 years to participate within ERS [2]; and, (c) articles dated before January 2011 for CV and MH disorders, but open search for MSK. The reason for limiting search for CV and MH is due to the systematic review conducted by Pavey, et al., when the most recent updated review was conducted. This review is an update on current research since 2011, to improve recommendations and to advance NICE policy recommendations.

## Results

The search strategy detected initially 763 potentially relevant articles within the literature search. With this, 137 duplicates were removed. From this, 78 articles were screened due to their titles and abstracts being relevant, with then a further 64 excluded for not meeting inclusion criteria. Reasons for exclusion at this point included if they did not have the main aims of prevention, observation, or treatment of the health condition. From this, 56 articles were selected for further analysis by reading each full text. Of those, 13 studies were selected for inclusion (Fig. 1: flow of studies through search and screening).

### Characteristics of studies and results

In Tables 1, 2 and 3, the characteristics and results from each of the studies are described and explained for CV, MH, and MSK disorders.

**Table 1:** Articles selected for review of ERS effects on (1) CV disorders

Study	Design	Comparison	Time points	N, age (mean, SD)	Disorder	Length weeks	Prescription	Measures	Effect	Outcomes
Anokye, et al. 2011 [42]	Decision analytic model, Quantitative	Retrospective	Completion	N = 701 40–60 years Mean age = 50 SD = n/a	Cardiovascular Mental health	12	Gym based exercise, 2× weekly	QLAY	⇑ 51–88% cost-effective	ERS is associated with modest increase in lifetime costs and benefits. Cost-effectiveness of ERS is highly sensitive to small changes in the effectiveness. ERS cost is subject to significant uncertainty mainly due to limitations in clinical effectiveness evidence base.
Duda, et al. 2014 [25]	RCT, Quantitative	ERS SDT (*N* = 184) vs. Standard ERS (*N* = 163)	Baseline 3 months 6 months	*N* = 347 30–65+ Mean = n/a SD = n/a	Cardiovascular Mental health	10–12	Gym based exercise, 2× weekly	7D PAR BP (mmHg) BMI (kg/m^2^) Weight (kg) HADS anxiety HADS depression	⇑^a^ 120 *** ⇔^a^ ⇓^a^ − 0.24* ⇓^a^ -0.77* ⇓^a^-0.24 ⇓^a^-0.47*	Standard ERS: No sig. Changes in BP, but reductions in weight and BMI (reduced sig. at 6 months compared to baseline). 3 months’ follow-up: increase of 187 min (from baseline) in self-reported moderate/vigorous PA. 6 months’ follow-up: increase of 120 min. Sig. reduction in HADS depression scores, no sig. Diff. in anxiety. SDT-ERS: 3 months’ follow-up: increase of 196 min in self-reported moderate/vigorous PA compared to baseline. Sig. improvements in HADS anxiety and depression scores. 6 months’ follow-up: No sig. Diff. from baseline to 6 months’ in BP, BMI or weight. Increase of 114 min in self-reported moderate/vigorous PA. Sig reduction in HADs anxiety and depression.
Edwards, et al. 2013 [24]	RCT, Quantitative	Between time points	Baseline 6 months 12 months	*N* = 798 16+ years Mean = n/a SD = n/a	Cardiovascular Mental health	16	Gym based exercise & exercise classes, 1–2 x weekly	EQ-5D Adherence	⇑^a^ ⇑^a^	Participants with risk of CHD, were more likely to adhere to the full programme than those with mental health conditions/combination of mental health and risk of CHD. Those living in areas of high deprivation were more likely to complete the programme. Results of cost-effectiveness analyses suggest NERS is cost saving in fully adherent participants. Adherence at 16 weeks was 62%.
Hanson, et al. 2013 [44]	Observational cohort study, Quantitative	Between time points	Baseline 12 weeks Completion	*N* = 2233 Mean = 53 SD = 15.9	Cardiovascular	24	Gym based exercise, 2× weekly	GLTEQ Adherence	⇑^a^*** ⇑^a^***	ERS was more successful for over 55 s, and less successful for obese participants. Completers increased PA at 24 weeks. Leisure site attended was a significant predictor of uptake and length of engagement. Uptake *n* = 181, 12-week adherence *n* = 968, 24-week adherence *n* = 777.
Littlecott, et al. 2014 [40]	RCT, Quantitative	Between time points and ERS vs. usual care	Baseline 6 months 12 months	*N* = 2160 16–88 years Mean = n/a SD = n/a	Cardiovascular Mental health	16	Group aerobic exercise sessions, 2× weekly	Adherence BREQ	⇑^a,^ ⇓^b^ ⇑^a^	Improved adherence and improved psychosocial outcomes. Significant intervention effects were found for autonomous motivation and social support for exercise at 6 months. No intervention effect was observed for self-efficacy. Greatest improvements in autonomous motivation observed among patients who were least active at baseline. Individuals with CHD risk in the control group participated in more PA per week than those in the intervention group with CHD risk factors.
Mills, et al. 2013 [45]	Observational cohort study, Mixed method	Prediction of completion	Baseline	*N* = 1315 31–68 years Mean = 54 SD 12.4	Cardiovascular	26	Group, 1-to-1, gym, studio, swimming, 1–2 x weekly	BP (mmHg) Body mass (kg) Adherence	⇓^a^ 1.87*** ⇓^a^ 3.541*** ⇑^a^	Increased confidence and self-esteem. Link between age and attendance. Increased age, increased likelihood of adherence. 57% completed scheme, 33% achieved weight loss, 49% reduced BP. Those with CVD, more likely to attend and adhere, compared to pulmonary disorders.
Murphy, et al. 2012 [41]	RCT, Quantitative	ERS vs. usual care	12 months	N = 2160 16–88 years Mean = 52 SD = 14.7	Cardiovascular (*N* = 1559) Mental Health (*N* = 522)	16	1-to-1, aerobic and resistance exercise, 1–2 x weekly	7D PAR Adherence HADS depression HADS anxiety	⇑^b^ 1.19* ⇑^a, b^1.46* ⇓^a^ − 0.71* ⇓^a^ − 0.54	Increase PA observed among those randomised to ERS intervention compared to usual care, and those referred with CHD only. For those referred for MH alone, or in combination with CHD, there were sig. Lower levels of anxiety/depression, but no effect on PA.
Rouse, et al. 2011 [46]	Exploratory, Quantitative	SDT theory based program	Baseline	N = 347 Mean = 50.4 SD = 13.51	Cardiovascular Mental Health	12	Gym based exercise sessions, 1× weekly	IOCQ BREQ-2 SVS HADS	⇑^a^ ⇑^a^ 0.24 ** ⇑^a^ 0.17 * ⇓^a^ **	Autonomy support increased intrinsic motivation. Autonomous motivation was positively associated with vitality and PA intentions. Those who scored high on HADS, had high scores for PA intentions. Regression analyses revealed that the effects of autonomy support on mental health and PA intentions differed as a function of who provided the support (offspring, partner or physician), with the offspring having the weakest effects. Autonomy support and more autonomous regulations led to positive mental health outcomes.
Tobi, et al. 2012 [43]	Retrospective, Quantitative	Adherers vs. non-adherers	13 weeks Completion	N = 701 Mean age = 46.4 SD = 13.85	Cardiovascular (n = 111) Musculoskeletal (orthopaedic *n* = 164) Mental health (n = 141) *Respiratory (n = 34) Other (n = 23) Metabolic (n = 228)*	20–26	1-to-1, aerobic and resistance exercise, 1–2 x weekly	Adherence (DV) BMI (kg/m^2^) BP (mmHg)	⇑^b^ ** ^-^ ^-^	Longer term schemes increased adherence. Longer-term adherence was found for increasing age and medical condition. For every 10-year increase in age, the odds of people continuing exercise increased by 21.8%. Participants referred with metabolic conditions were more likely to adhere than those with orthopaedic, CV and other disorders. Longer-term schemes offer the opportunity to maintain adherence to exercise.
Webb, et al. 2016 [47]	Evaluation, Quantitative	NERS vs. community-based exercise vs. continuously monitored exercise programme	Baseline Completion	*N* = 107 Mean = 44.6 SD = 11.4	Cardiovascular	8	Group exercise sessions, 2× weekly	IPAQ (min/week) BMI (kg/m^2^) Systolic BP (mmHg) Diastolic BP (mmHg) Adherence	⇑^a, b^ 540*** ⇓^a, b^ 0.4 + 0.1** ⇓^a, b^ -6.1 + 2.6* ⇓^a^ -0.6 + 1.8 ⇑^b^ *	CV health benefits were observed in all three interventions. CV health benefits achieved in laboratory based studies were achieved in ERS settings. BMI had bigger reductions in NERS compared to the other two conditions. Systolic BP and Diastolic BP were also reduced more in NERS compared to the other two conditions.

^a^all comparisons are with baseline value -not available in the results

^b^all comparisons are with control ***p < 0.001, ** *p* < 0.01, * p < 0.0

*CVD* cardiovascular disease, *CHD* coronary heart disease, *QALY* quality adjusted life-year, *7D PAR* 7-day physical activity recall scale, *IPAQ* international physical activity questionnaire, *BMI* body mass index, *BP* blood pressure, *HADS* hospital anxiety and depression scale, *EQ-5D* EuroQol 5 dimension, *GLTEQ* Godin leisure-time exercise questionnaire, *BREQ*-behavioural regulation in exercise questionnaire, *SVS* subjective vitality scale, *IOCQ* important other climate questionnaire

⇓= reductions in scores, ⇑ = increase in scores, ⇔ no change

**Table 2:** Articles selected for review of ERS effects on (2) MH disorders

Study	Design	Comparison	Time points	N, age (mean, SD)	Disorder	Length (weeks)	Prescription	Measures	Effect	Outcomes
Anokye, et al. 2011 [42]	Decision analytic model, Quantitative	Retrospective	Completion	N = 701 40–60 years Mean age = 50 SD = n/a	Mental health Cardiovascular	12	Gym based exercise, 2× weekly	QLAY	⇑ 51–88% cost-effective	ERS is associated with modest increase in lifetime costs and benefits. Cost-effectiveness of ERS is highly sensitive to small changes in the effectiveness. ERS cost is subject to significant uncertainty mainly due to limitations in clinical effectiveness evidence base.
Chalder, et al. 2012 [48]	RCT, Quantitative	ERS vs. usual care	Baseline 4 months 8 months 12 months	*N* = 361 18–69 years Mean = 40.9 SD = 12.5	Mental health	8	Group aerobic exercise classes, 1-4× weekly	BDI 7D PAR	⇓^a, b^ -0.54 *p* = 0.68 ⇑^a, b^ *p* = 0.08	Increased PA, improved mood. No reduction in antidepressant use in ERS group. A mean 7.2 (SD 4.1) sessions was completed. More people reported increased PA at the follow up in ERS, than those in usual care.
Duda, et al. 2014 [25]	RCT, Quantitative	ERS SDT (N = 184) vs. Standard ERS (N = 163)	Baseline 3 months 6 months	N = 347 30–65+ Mean = n/a SD = n/a	Mental health Cardiovascular	10–12	Gym based exercise, 2× weekly	7D PAR BP (mmHg) BMI (kg/m^2^) Weight (kg) HADS anxiety HADS depression	⇑^a^ 120 *** ⇔^a^ ⇓^a^ − 0.24* ⇓^a^ -0.77* ⇓^a^ -0.24 ⇓^a^-0.47*	Standard ERS: No sig. Changes in BP, but reductions in weight and BMI (reduced sig. at 6 months compared to baseline). 3 months’ follow-up: increase of 187 min (from baseline) in self-reported moderate/vigorous PA. 6 months’ follow-up: increase of 120 min. Sig. reduction in HADS depression scores, no sig. Diff. in anxiety. SDT-ERS: 3 months’ follow-up: increase of 196 min in self-reported moderate/vigorous PA compared to baseline. Sig. improvements in HADS anxiety and depression scores. 6 months’ follow-up: No sig. Diff. from baseline to 6 months’ in BP, BMI or weight. Increase of 114 min in self-reported moderate/vigorous PA. Sig reduction in HADs anxiety and depression.
Edwards, et al. 2013 [24]	RCT, Quantitative	Between time points	Baseline 6 months 12 months	*N* = 798 16+ years Mean = n/a SD = n/a	Mental health Cardiovascular	16	Gym based & exercise classes, 1–2 x weekly	EQ-5D Adherence	⇑^a^ ⇑^a^	Participants with risk of CHD, were more likely to adhere to the full programme than those with mental health conditions/combination of mental health and risk of CHD. Those living in areas of high deprivation were more likely to complete the programme. Results of cost-effectiveness analyses suggest NERS is cost saving in fully adherent participants. Adherence at 16 weeks was 62%.
Littlecott, et al. 2014 [40]	RCT, Quantitative	ERS (N = 1080) vs. control (*N* = 1080)	Baseline 6 months 12 months	N = 2160 16–88 years Mean = n/a SD = n/a	Mental health Cardiovascular	16	Group aerobic exercise sessions, 2× weekly	Adherence BREQ	⇑^a,^ ⇓^b^ ⇑^a^	Improved adherence and improved psychosocial outcomes. Significant intervention effects were found for autonomous motivation and social support for exercise at 6 months. No intervention effect was observed for self-efficacy. Greatest improvements in autonomous motivation observed among patients who were least active at baseline. Individuals with CHD risk in the control group participated in more PA per week than those in the intervention group with CHD risk factors.
Murphy, et al. 2012 [41]	RCT, Quantitative	ERS vs. usual care	12 months	N = 2160 16–88 years Mean = 52 SD = 14.7	Mental Health (N = 522) Cardiovascular (N = 1559)	16	1-to-1, aerobic and resistance exercise, 1–2 x weekly	7D PAR Adherence HADS depression HADS anxiety	⇑^b^ 1.19* ⇑^a, b^ 1.46* ⇓^a^ − 0.71* ⇓^a^ − 0.54	Increase PA observed among those randomised to ERS intervention compared to usual care, and those referred with CHD only. For those referred for MH alone, or in combination with CHD, there were sig. Lower levels of anxiety/depression, but no effect on PA.
Rouse, et al. 2011 [46]	Exploratory, Quantitative	SDT theory based program	Baseline	N = 347 Mean = 50.4 SD = 13.51	Mental Health Cardiovascular	12	Gym based exercise sessions, 1× weekly	IOCQ BREQ-2 SVS HADS	⇑^a^ ⇑^a^ 0.24 ** ⇑^a^ 0.17 * ⇓^a^ **	Autonomy support increased intrinsic motivation. Autonomous motivation was positively associated with vitality and PA intentions. Those who scored high on HADS, had high scores for PA intentions. Regression analyses revealed that the effects of autonomy support on mental health and PA intentions differed as a function of who provided the support (offspring, partner or physician), with the offspring having the weakest effects. Autonomy support and more autonomous regulations led to positive mental health outcomes.
Tobi, et al. 2012 [43]	Retrospective, Quantitative	Adherers vs. non-adherers	13 weeks Completion	N = 701 Mean = 46.4 SD = 13.85	Mental health (*n* = 141) Musculoskeletal (orthopaedic *n* = 164) Cardiovascular (*n* = 111) *Respiratory (n = 34) Other (n = 23) Metabolic (n = 228)*	20–26	1-to-1, aerobic and resistance exercise, 1–2 x weekly	Adherence (DV) BMI (kg/m^2^) BP (mmHg)	⇑^b^ ** ^-^ ^-^	Longer term schemes increased adherence. Longer-term adherence was found for increasing age and medical condition. For every 10-year increase in age, the odds of people continuing exercise increased by 21.8%. Participants referred with metabolic conditions were more likely to adhere than those with orthopaedic, CV and other disorders. Longer-term schemes offer the opportunity to maintain adherence to exercise.

*CVD* cardiovascular disease, *CHD* coronary heart disease, *BDI* Beck depression inventory, *QALY* quality adjusted life-year, *7D PAR* 7-day physical activity recall scale, *IPAQ* international physical activity questionnaire, *GPPAQ* general practice physical activity questionnaire, *BMI* body mass index, *BP* blood pressure, *HADS* hospital anxiety and depression scale, *EQ-5D* EuroQol 5 dimension, *GLTEQ* Godin leisure-time exercise questionnaire, *BREQ*-behavioural regulation in exercise questionnaire, *SVS* subjective vitality scale, *IOCQ* important other climate questionnaire

⇓= reductions in scores, ⇑ = increase in scores, ⇔ no change

^a^all comparisons are with baseline value

^b^all comparisons are with control

-not available in the results

***p < 0.001, ** *p* < 0.01, * *p* < 0.05

**Table 3:** Articles selected for review of ERS effects on (3) MSK disorders

Study	Design	Comparison	Time points	N, age (mean, SD)	Disorder	Length (weeks)	Prescription	Measures	Effect	Outcomes
Hillsdon, et al. 2002 [49]	RCT, Quantitative	ERS vs. no intervention	Baseline 12 months	*N* = 1658 45–64 years Mean = n/a SD = n/a	Musculoskeletal	12	1-to-1 exercise sessions, weekly	Self-reported PA MLTAQ BMI (kg/m^2^) Systolic BP (mmHg) Diastolic BP (mmHg)	⇑ 124, *p* = 0.39 ⇑ ⇓ *p* = 0.86 ⇓ *p* = 0.81 ⇓**	Intention to treat analysis revealed no significant differences in PA between groups. Community-based PA ERS have some impact on reducing sedentary behaviour in the short-term, but unlikely to be sustained and lead to benefits in terms of health.
James, et al 2009 [50]	Observational cohort study Quantitative	Population based analysis	Completion	N = 1315 Under 50 = 539 Over 50 = 776 Mean = n/a SD = n/a	Musculoskeletal	26	1-to-1 and group exercise sessions	BMI (kg/m^2^) BP(mmHg)	⇓1.292 *p* = 0.043 ⇓*	Completers demonstrated an increased likelihood of reduced BP. Participants who achieved a reduction in body mass had an increased likelihood of achieving reduced BP. Completion is associated with reduced body mass and BP.
Tobi, et al. 2012 [43]	Retrospective, Quantitative	Adherers vs. non-adherers	13 weeks Completion	*N* = 701 Mean = 46.4 SD = 13.85	Musculoskeletal (orthopaedic *n* = 164) Cardiovascular (n = 111) Mental health (n = 141) *Respiratory (n = 34) Other (n = 23) Metabolic (n = 228)*	20–26	1-to-1, aerobic and resistance exercise, 1–2 x weekly	Adherence (DV) BMI (kg/m^2^) BP (mmHg)	⇑^b^ ** ^-^ ^-^	Longer term schemes increased adherence. Longer-term adherence was found for increasing age and medical condition. For every 10-year increase in age, the odds of people continuing exercise increased by 21.8%. Participants referred with metabolic conditions were more likely to adhere than those with orthopaedic, CV and other disorders. Longer-term schemes offer the opportunity to maintain adherence to exercise.

CVD cardiovascular disease, CHD coronary heart disease, IMD index of multiple deprivation, MLTAQ Minnesota leisure time activity questionnaire, 7D PAR 7-day physical activity recall scale, BMI body mass index, BP blood pressure, HADS hospital anxiety and depression scale, EQ-5D EuroQol 5 dimension, GLTEQ Godin leisure-time exercise questionnaire, BREQ-behavioural regulation in exercise questionnaire, SVS subjective vitality scale, IOCQ important other climate questionnaire

⇓= reductions in scores, ⇑ = increase in scores, ⇔ no change

^a^all comparisons are with baseline value

^b^all comparisons are with control

-not available in the results

***p < 0.001, ** *p* < 0.01, * *p* < 0

### Results for cardiovascular disorders’ sub-group

All ten articles collected quantitative data. Four randomised controlled trials (RCTs) were included in the review for CV disorders. One RCT compared standard ERS to a self-determination theory (SDT)-based ERS [25], two RCTs compared ERS to usual care [40, 41], and one compared to baseline [24]. A retrospective study reviewed the costs and benefits associated with ERS [42]. Another study compared the characteristics of adherers and non-adherers [43]. One observational cohort study [44] reviewed outcomes between time points: baseline, 6 months, and 12 months, whilst another examined the success of ERS in order to predict completion [45]. Another study used an exploratory approach to review the role of autonomy support upon entering ERS [46]. Whilst a final paper conducted an evaluation of ERS compared to community-based exercise and a continuously-monitored exercise programme [47].

### CV disorder-related outcomes

Four studies contained participants with coronary heart disease (CHD) or who were at increased CHD risk [24, 25, 40, 41]; six studies with CV disease or at increased CV disease risk [41, 43, 45–47]; and one study included participants with hypertension [42]. Even though various designs were employed, body mass index (kg/m^2^) or body mass decreased [25, 45, 47] (no results of BMI in one study [43]), with one study showing a significant difference of 0.24 kg/m^2^ (*p* < 0.05) compared to baseline [25]. Webb, et al. [47] found reductions in BMI compared to baseline and control. Mills, et al. [45] did not report on BMI, but did report on body mass, finding significant reductions in body mass (*p* < 0.001) compared to baseline, with 33.3% of participants achieving weight loss.

Both systolic and diastolic Blood pressure was recorded in four studies [25, 43, 45, 47]. Mills, et al. [45] found significant reductions in BP compared to baseline (*p* < 0.001), with 49% of participants reducing BP. Webb, et al. [47] reported significant reductions in systolic BP and diastolic BP. Tobi, et al. [43] did not report on BP findings. BP was not reduced, compared to baseline within one study [25].

BMI was also reported to have significantly reduced at 6 months, compared to baseline, however clinically, this reduction was only a small amount [25].

### Physical activity outcomes

Compared to baseline, two of the four RCTs showed increased 7-day physical activity recall (7D PAR) scores [25, 41], with two of these studies reporting significant increases (*p* < 0.001). One study showed a significant increase (*p* < 0.05) compared to control [41]. Murphy, et al. [41] found significant increase in 7D PAR compared to usual care. Standard ERS compared to a SDT-based ERS found that both schemes significantly improved self-reported PA, with no significant difference between the two conditions [25].

### Adherence

Seven out of ten studies recorded PA adherence to the ERS programme. Compared to baseline, adherence to PA prescribed increased in every study. Hanson, et al. [44] reported significant differences (*p* < 0.001) along with Murphy, et al. [41] and Webb, et al. [47] showing significant differences of *p* < 0.05, and Tobi, et al. [43] also having significant difference (*p* < 0.01). Littlecott, et al. [40] found increased adherence to the prescription compared to baseline, but not compared to usual care.

### Duration and mode/type of exercise

Scheme’s duration was either 8–12 weeks [25, 42, 46, 47], 16 weeks [24, 40, 41] or 20–26 weeks [43–45], consisting of a prescription of 1–2 exercise sessions per week. Nine studies utilised one-to-one gym based PA as their main mode of exercise, consisting of both cardiovascular and resistance training activities. Two studies utilised group based aerobic exercise sessions [45, 47], along with other forms of PA including swimming and PA within studio settings [45].

### Results for mental health disorders’ sub-group

Five of the eight studies were RCTs [24, 25, 40, 41, 48]. Three of these compared ERS to usual care [40, 41, 48], one compared ERS to a SDT-based ERS [25], or between time points [24]. A further study used a SDT theory-based exploratory design [46]. A retrospective study compared adheres to non-adheres [43].

Anokye, et al. [42] reviewed the cost-effectiveness of ERS overall. It was found that ERS was linked to a slower increase in lifetime costs and benefits. ERS was found to be 51–88% cost-effective. However, this was not only related to mental health, but generic ERS.

### MH disorder-related outcomes

Seven studies conducted research on individuals diagnosed with either mild or moderate depression/anxiety/stress [24, 25, 40–42, 46, 48], with one study not giving a detailed description of the mental health disorder [43].

Various psychosocial/psychological measures were employed, with the Hospital Anxiety and Depression Scale (HADS) being the most common. HADS anxiety and depression were both decreased compared to baseline [25, 41]. HADS depression showed lower scores compared to HADS anxiety, with significant differences between changes (*p* < 0.05) [25, 41]. Chalder, et al. [48] reported decreases in scores on the Beck Depression Inventory, compared to both baseline and control. Behavioural Regulation in Exercise Questionnaire (BREQ) scores also increased compared to baseline, with one study showing significant increase in results at completion (*p* < 0.01) and Subjective Vitality Scale (SVS) scores also showing significant increases at completion (*p* < 0.05) [46].

### Physical activity outcomes

7D-PAR scores reported increases in PA at completion compared to baseline [22, 25], and compared to control [41, 48]. Significant increases (*p* < 0.001, *p* < 0.05) were found in three of these studies [25, 41, 48].

### Duration and mode/type of exercise

Scheme’s duration was either 8 weeks [48], 10–12 weeks [25, 42, 46], 16 weeks [24, 40, 41] or 20–26 weeks long [43], prescribing 1–2 exercise sessions weekly. Seven schemes used one-to-one gym based PA, consisting of both cardiovascular and resistance training activities.

Two studies utilised group based aerobic exercise sessions [45, 47], along with other forms of PA including swimming and PA within studio settings [45].

### Results for musculoskeletal disorders’ sub-group

Research reviewing MSK disorders is extremely limited. Within a complete search, only three articles showed some relevance to the disorder, although, MSK measures were not used. All three studies facilitated different designs which included a RCT [49], an observational cohort study [50], and a retrospective design [43].

### MSK disorder-related outcomes

While these papers include referrals with MSK disorders, uptake is low, along with no MSK disorder-specific measures.

### Physical activity outcomes

Adherence to longer length schemes was better than shorter length schemes [43]. When reported using an objective measure of adherence to the PA prescribed, rather than subjective self-reported PA, longer schemes had a significant difference on adherence to PA significant increase *p* < 0.01) [43]. It was also suggested in the same study that for every 10-year increase in age, odds of exercise continuation increased by 21.8%.

### Duration and mode/type of exercise

One study’s length was 12 weeks [51], with two studies of 20–26 weeks in length [49, 52]. They all utilised one-to-one cardiovascular and resistance exercise with a prescription of 1–2 sessions weekly. Additionally, one study also incorporated group exercise sessions alongside the one-to-one sessions [50].

## Discussion

### Summary of findings

The aim of this review was to examine the effects of ERS within three populations: those with CV, MH, and MSK disorders. Length of schemes, and mode and type of exercise used with each sub-group, was reviewed due to inconsistencies in previous research [2]. At present, strong research is lacking to support ERS of 12-week duration which are recommended by NICE [22], particularly with respect to adherence to PA prescribed; evidence suggests schemes do not tailor the mode and type of exercise specifically to suit health disorders; and the evidence to support ERS in specific disorders in relation to adherence and improving health outcomes is poor [2, 25, 28]. These two key variables will be discussed further in detail.

Schemes have evidenced the effectiveness in CV [25, 43, 47] and MH disorders [25, 41], but evidence is lacking around MSK disorders. Duration and type of ERS were elements to consider in terms of their impact on outcomes. Overall, ERS resulted in significant reductions in BP [45, 47, 49, 50] and BMI [25, 47, 49, 50], and increased adherence to the PA prescribed over time [24, 40, 41, 43, 44, 49]. Self-reported PA levels also increased [25, 41, 44, 49]. Prior to this review, a previous systematic review suggested that separately reported health outcomes relating to referral reason have not been reported [2]. Within this review, the health disorder sub-groups were individually analysed to review any disorder-specific outcomes.

Within the CV sub-group, not all studies reported a disorder-specific measure [24, 40–42, 44, 46]. To get a true representation of any improvements made in the CV sub-group, all studies should have reported on the disorder-specific measures. Of those who did report on CV-specific measures, all showed improvements in BP and BMI [25, 45, 47].

Individuals referred for MH disorders, responded positively to either gym based exercise sessions or group aerobic exercise sessions. Disorder-specific measures such as HADS showed that ERS significantly reduced anxiety and depression scores [25, 41]. These were self-reported measures of anxiety and depression. Other measures were also reported, but most individuals referred tend to have more than one health disorder [22], which can be observed in the articles reviewed in Tables 1, 2 and 3.

MSK disorders have limited research of the effects of ERS on disorder-specific outcomes. There are no direct measures used to evaluate the effects of ERS on the MSK disorders (such as measuring pain felt in the injured area/range of movement/functional outcomes). Using measures such as Lower Extremity Functional Scale (LEFS), Lower Limb Functional Index (LLFI) [53], McGill Pain Questionnaire (MPQ) [52] or the Visual Analogue Scale [54] could be tools which could more accurately measure MSK disorders. At present one in five people consult a GP about MSK pain each year. Support and treatment for MSK chronic pain account for approximately 4.6 million appointments per year [51]. If further research into the effectiveness of ERS on MSK disorders was conducted then, if effective, GP time could be reduced, saving time and money for the NHS.

### Length of schemes

ERS tend to conclude after a 10–12-week exercise programme within England and Ireland [2, 23], although longer length schemes offer more opportunity for individuals to gain long term health benefits of PA [43, 44, 50]. NICE [22] who set out the guidelines for ERS, recommend schemes last for at least 12 weeks. Research regarding longer length schemes is extremely limited. However, a previous study suggests that longer length schemes have been beneficial for individuals with CV disease risk and MH disorders, increasing PA levels whilst also being more cost-effective [24]. Research relating to 12-week ERS suggests that significant health outcomes and changes in PA do not occur [27]. Many studies likely have employed 12-week schemes to meet the guidelines set out by NICE [22]. However, if ERS scheme’s length recommendations were increased by NICE, then it might be expected that the implementation of ERS schemes would follow suit and thus greater effects on health outcomes might occur.

Shorter-length ERS (8–10 weeks) did not produce the same outcomes as schemes of longer lengths. For example, a scheme of short length (8 weeks) did not have statistically significant effects on physiological and psychosocial outcomes for individuals referred for MH disorders [48]. However, Webb, et al. [47] did find significant changes in BP, through an 8-week long scheme for participants referred with CV disorders, although longer length schemes produced better outcomes [43, 45]. Thus, it could be argued that shorter length schemes should have the potential to impact CV disorders, though longer schemes may be required for MH disorders. Past research has showed that exercise can have positive impacts on CV outcomes after only a couple of weeks of participating in 30-min of regular vigorous exercise [55]. Further research supports that four weeks of aerobic and resistance exercise can improve blood pressure, arterial stiffness and blood flow [56, 57]. It has also been reported that diastolic and systolic blood pressure can be reduced after one exercise session, and remain low for up to 90 min’ post-exercise session [58–60]. Thus, could support the use if 8-week ERS for participants with CV conditions, in the improvement of CV-specific health outcomes such as systolic and diastolic blood pressure.

Mid length schemes (11–19 weeks) did show significant improvement in the conditions examined. As noted, NICE [22] schemes have stated that schemes should be at least 12 weeks in length. Various other clinical and traditional exercise programmes are often longer than 12 weeks [24, 61–63] and demonstrate greater efficacy for improving health conditions [47]. Therefore, ERS guidelines should perhaps be adapted to match this. Compared with an 8-week ERS, Webb, et al. [47] found that within an 8-week community-based outdoor exercise programme, participants achieved higher intensities of effort resulting in pronounced beneficial effects on health including: significant CV disease risk-lowering; reduced blood pressure; arterial stiffness; and blood lipids. Increasing the length of ERS may permit them to produce results more comparable with other exercise and PA interventions. Further, consideration of the mode of exercise, could improve the effectiveness of ERS. To support this, Duda, et al. [25] found that there were no significant changes in BP in schemes of 10–12 weeks in length. This may be due to other cofounding influences which may have affected blood pressure not allowing it to reduce in the short term, including medication [64]. However, combinations of longer duration exercise interventions with medication may potentially provide a more stable and positive effect on blood pressure [65].

Studies of schemes following the NICE [22] recommended length of 12 weeks found that, compared to no intervention, self-reported PA levels did not differ [49]. This could suggest that 12 weeks is not long enough to initiate changes in PA which are perceivable by participants. Longer length schemes may improve self-reported PA. However, schemes of this length had some impact on reducing sedentary behaviours, but it was suggested that this was unlikely to be sustained and lead to long term health benefits such as weight loss, sustained reduced BP, and decreased BMI [49].

Longer-length schemes (20+ weeks) have been shown to be beneficial in improving various health outcomes and aid healthier behaviours [7, 45, 50]. All longer-length schemes reviewed had positive impacts on health, reducing BP and BMI [26], improving PA levels [24] and increasing adherence to the prescription [43–45, 50]. At present, guidelines use 12-week ERS as a basis for providers to follow [22] whereas a change in guidelines to introduce longer length schemes might result in more providers delivering ERS of such length in the UK and produce better health outcomes as well as cost savings for the NHS [24].

### Type and mode of exercise

The most common type of exercise employed in ERS was one-to-to one supervised gym based exercise sessions, incorporating both resistance and cardiovascular exercise for all health conditions [24, 25, 41, 43, 44, 46]. Individuals referred for CV disorders, who incorporate both resistance and aerobic exercises into their prescription, saw greater improvements in CV health which is supported by past research [66, 67]. This is in line with results found within studies included in this review [25, 45, 47].

MH disorders also improved significantly when individuals took part in aerobic and resistance training gym based exercise. Scores relating to depression and anxiety had all improved [24, 25, 41, 43, 46]. Physical activity levels had also increased. This could suggest that gym based exercise sessions incorporating aerobic and resistance exercise are best suited in reducing MH disorders. Indeed, a recent meta-analysis supports the use of resistance exercise in treatment of anxiety [12] while previous reviews also support the benefits of aerobic training [68]. Both are clearly effective, yet may exert specific effects upon MH outcomes. Thus, the combined approach may be best suited for ERS in MH disorders.

There is very limited research on ERS with MSK disorders, therefore it is difficult to compare the results from this review to past literature. Only three articles were found to be relevant for this review for this population [46, 49, 50]. All comprised of predominantly one-to-one exercise sessions, and all reported increases in adherence to PA prescribed across time. Unfortunately, none of the studies included any outcomes related to the patient’s MSK disorders such as pain or disability. Considering that all also utilised similar interventions it is therefore difficult to discern specifically the comparative efficacy of different types of ERS in MSK disorders. However, there is evidence to suggest that, similarly to other disorders, using both aerobic and resistance exercises do improve musculoskeletal disorders including osteoarthritis of the knee [69].

Aerobic exercise sessions were solely the mode of some schemes [40, 48]. However, as has been shown, prescriptions of exercise that solely focus on aerobic exercise may be less efficacious as combined approaches. Resistance training exerts a wide range of benefits alongside aerobic training [70, 71]. Additionally, aerobic exercise-only ERS present its own issues such as lack of efficacy as typically employed in reducing or stopping lean body mass loss, and associated loss in resting metabolic rate per decade affiliated with normal ageing [72]. That the majority of research has focused upon the health benefits gained from aerobic training has made this mode of exercise a primary focal point within PA guidelines according to literature [73]. However, it has been argued that resistance training based interventions should have a greater emphasis in public health approaches [74].

At present NICE guidelines [22] do not advise on the type and mode of exercise that should be employed within ERS, though the majority of studies here show that one-to-one gym based exercise sessions employing both aerobic and resistance training are effective. Tailoring the type and mode of exercise to be disorder-specific could also influence adherence and health outcomes. The evidence reviewed here suggests that a combining both aerobic and resistance exercise is effective across a range of disorders. However, there is a lack of research directly comparing different ERS utilising different exercise approaches. Some individuals may also be referred for multiple disorders, and this may need an entirely different approach. Usually, a referral is made for one health disorder, but if an individual is referred for more than one disorder, then a more nuanced exercise programme may be required. This may also mean that the scheme’s length needs adjusting to suit the amount of disorders referred for. Further research is required to analyse the type and mode of exercise prescribed dependant on the disorders and health outcomes upon completion.

### Implications for future research and clinical practice

The usual length of schemes in most ERS is 12 weeks long [22]. Research within this review has found that longer schemes (20 weeks+) may provide better effects on adherence to the prescription and health outcomes [43, 44, 50]. This conclusion suggests that recommendations set out by NICE [22] might benefit from being updated to emphasise the importance of longer schemes. Indeed, as noted, longer schemes have also been shown to be more cost effective [24]. A key challenge for future research is to identify ways to maximise uptake and improve adherence to PA prescribed until completion across all schemes.

At present, ERS are not meeting several standards set out by NICE including: referral of individuals who are sedentary/inactive but otherwise healthy; incorporate behaviour change into individual approaches; agreeing goals and sticking to action plans with regular follow ups with no-shows; and tailoring the intervention to individual needs and develop coping plans to prevent relapse. At present, though often one-to-one sessions are employed, schemes are typically generic and not personalised to suit individuals and their health disorders specifically. One-to-one gym based exercise sessions can potentially be tailored to individual needs of each participant and health disorder. However, within this review, there was no information given within studies on how programmes were tailored to suit each participant, or if they were at all. At present, NICE [22] have not set any guidelines on the type and mode of exercise which is to be administered, let alone disorder-specific exercise guidelines. Broadly the results of this review suggest that combined approaches of both cardiovascular and resistance exercises are effective across disorders. Yet there is little research directly comparing different approaches, or comparing generic interventions to those with specific individualisation. By tailoring programmes to suit each patient, ERS could address some of the barriers which some patients report stop them from adhering to schemes, including unfamiliar environment, quality of interaction with exercise provider, boredom, exercise preferences, poor record keeping, and clinical disorder [21].

Economic impact of ERS was reported in one study [42]. Results show that for sedentary individuals with CVD, and sedentary individuals with a MH disorder, the estimated cost per quality-adjusted life year (QALY) was £12,834 and £8414 respectively. Benefits and incremental lifetime costs linked with ERS were found to be sensitive to variations in the relative risk of ERS costs and becoming physically active. ERS is more expensive compared to usual care, due to the additional mean lifetime costs of £170 per individual, although, it is more effective in leading to a lifetime mean QALY gain of 0.008 per individual.

Although schemes need to be cost-effective, future training of exercise referral instructors could be adapted to improve exercise prescriptions with updated evidence-based guidelines. This may reduce the burden of cost on ERS, as instructors will be more equipped to prescribe exercise which may have greater effects on health outcomes. An evaluation of a scheme in Belfast found that they calculated a return of approximately £7 for every £1 invested into their Healthwise Physical Activity Referral Programme [75]. Further, Anokye, et al. [42] reviewed the cost-effectiveness of ERS. It was found that ERS was linked to a slower increase in lifetime costs and benefits. ERS was found to be 51–88% cost-effective.

Other identified issues within this review include that control interventions are often not explained in detail [25, 40, 43, 48, 50, 64]. They must distinctly differ from ERS, and be explained in detail, in order to examine comparative effectiveness. Another identified issue, which may also provide evidence for NICE policies is that health-economic evaluations are often not incorporated into studies to review the cost-effectiveness of the schemes alongside the effectiveness on health outcomes. Evidence of cost-effectiveness is also required to understand the wider benefits of GPs referring patients to ERS reducing burdens on the NHS.

## Conclusion

The current review present an updated overview of ERS in the UK and provides insights to aid in guideline revisions and policy development, in addition to identifying area’s where research is still required. It can be concluded that longer length schemes may produce more significant positive effects on health outcomes than shorter schemes. These effects include reduced BP and BMI [45, 50], improved physical activity levels [7] and increasing adherence to the PA prescribed over time [43–45, 50]. At present, NICE [22] recommend schemes last for 12 weeks, but it appears that this guidance should be updated to increase length of schemes. This in turn may improve adherence to exercise and physical activity. The gap between research and policy needs to be bridged. Further research which is required to examine the comparative effects of specific types and modes of exercise, and in the improvement of specific health disorders. Such evidence might help produce predictive models to allow GPs to identify the best referral pathways for patients. Alongside a predictive model, an economic evaluation of ERS, compared to usual care specifically designed around each disorder type, could also help to inform policy decisions.

## Abbreviations

7D PAR: 7-day physical activity recall scale
BMI: Body mass index
BP: Blood pressure
BREQ: Behavioural regulation in exercise questionnaire
CHD: Coronary heart disease
CV: Cardiovascular
CVD: Cardiovascular disease
EQ-5D: EuroQol 5 dimension
ERS: Exercise referral schemes
GLTEQ: Godin leisure-time exercise questionnaire
GP: General practitioner
HADS: Hospital anxiety and depression scale
IMD: Index of multiple deprivation
IOCQ: Important other climate questionnaire
MH: Mental health
MLTAQ: Minnesota leisure time activity questionnaire
MSK: Musculoskeletal
NHS: National Health Service
PA: Physical activity
PIA: Physical inactivity
QALY: Quality-adjusted life year
RCT: Randomised controlled trial
SVS: Subjective vitality scale
UK: United Kingdom
